# Preclinical study to improve microbubble-mediated drug delivery in cancer using an ultrasonic probe with an interchangeable acoustic lens

**DOI:** 10.1038/s41598-021-92097-z

**Published:** 2021-06-16

**Authors:** Seunghyun Lee, Hoyoon Jeon, Shinyong Shim, Maesoon Im, Jinsik Kim, Jung Hoon Kim, Byung Chul Lee

**Affiliations:** 1 Department of Radiology, Seoul National University Hospital, 101 Daehak-ro, Jongno-gu, Seoul, 03080 Republic of Korea; 2 Department of Radiology, Seoul National University College of Medicine, Seoul, 03080 Republic of Korea; 3 Institute of Radiation Medicine, Seoul National University Medical Research Center, 103 Daehak-ro, Jongno-gu, Seoul, 03080 Republic of Korea; 4 Brain Science Institute, Korea Institute of Science and Technology (KIST), Seoul, 02792 Republic of Korea; 5 Department of Medical Biotechnology, Dongguk University, Seoul, 04620 Republic of Korea

**Keywords:** Cancer, Drug discovery, Diseases, Medical research, Oncology, Engineering

## Abstract

Focused ultrasound with microbubbles (FUS-MBs) has shown that it can lead to an efficient drug delivery system (DDS) involving the oscillation and destruction of the MB but is limited in drug delivery due to its narrow pressure field. However, unfocused ultrasound with MBs (UUS-MBs) and an interchangeable acoustic lens can tune and enhance the pressure field for MB destruction to overcome the disadvantages of FUS-MB DDSs. We designed a lens suitable for an ultrasound-phased array probe and studied the optimal treatment conditions for MB destruction in vitro through an optical imaging setup. The DDS effects were evaluated in a rat hepatoma model using doxorubicin (DOX) treatment. A concave lens with a radius of curvature of 2.6 mm and a thickness of 4 mm was selected and fabricated. UUS-MBs with the acoustic lens at 60 V_pp_ for 32 cycles and a PRF of 1 kHz could induce MB destruction, promoting the DDS even under fluidic conditions. In the animal experiment, the UUS-MBs in the acoustic lens treatment group had a higher concentration of DOX in the tumor than the control group. Our system suggests uses an acoustic lens to increase DDS effectiveness by providing sufficient ultrasound irradiation to the MBs.

## Introduction

Drug delivery systems (DDSs), which can enhance therapeutic effects, have gained considerable interest as potential noninvasive adjuvant treatments^1–4^. Chemotherapy, a type of cancer treatment using a drug, has been adopted to overcome the difficulty of removing metastasized cancer through surgery. However, conventional cytotoxic agents such as doxorubicin (DOX), which is a widely used chemotherapeutic agent, have low response rates and high complication rates with respect to their cytotoxicity to normal tissues^5,6^. Therefore, it is crucial to improve drug delivery to a specific cancer site without increasing the dose to normal cells and tissues. As drug delivery strategies^7–9^, mechanical force-triggered DDSs have been approached as a means of releasing or activating the drug at the target site. Ultrasound has significant potential for treating cancer with the capability to focus on specific target sites inside the body with fewer side effects.

Focused ultrasound with microbubbles (FUS-MBs), which are commonly used in clinical practice due to their proven safety, have shown highly efficient therapeutic effects with complex phenomena that involve the oscillation and destruction of MBs^10,11^. Recent research has shown improvements in cancer drug delivery to malignant tumors with FUS-MBs^12,13^. Despite these promising results, fixed-focused ultrasound with a narrow pressure field might limit accurate drug delivery by aligning blood vessels to the lesion due to the vascular structure of cancer. Cancer reconstructs the appearance of blood and lymphatic vessels in extraordinary ways that are not confined to the initial disease area. These unpredictable vascular structures have limitations with respect to irradiating blood vessels using focused ultrasound with a narrow focal point. In this case, a monitoring system for finding the focal target spot is necessary^14,15^. Thus, a complex FUS-MB DDS requires an ultrasonic array transducer, front-end electronics to control each array element, and other medical imaging systems for monitoring the spots being stimulated. In addition, thermal damage due to the high energy of focused ultrasound is an inevitable problem^16,17^.

Previous studies have been employed to address these limitations via unfocused ultrasound with MBs (UUS-MBs), and the effectiveness of UUS-MBs has also been demonstrated. For example, Beccaria et al.^18^ and Yao et al.^19^ published papers reporting that UUS-MBs could effectively deliver drugs to an extensively targeted area. However, Kovacs et al. asserted that tumor migration, not observed with FUS-MBs, was derived from drug delivery in an extensive area^20^. Thus, for drug delivery with UUS-MBs, the prevention of unexpected damage caused by widespread excessive ultrasonic irradiation is essential. Unlike conventional unfocused transducers, ultrasound with an acoustic lens can tune the pressure field depending on the type of interchangeable acoustic lens according to the purpose of the DDS^21,22^. Simple attachment of the inexpensive lens on the purchased diagnostic ultrasound probe can also reduce the tremendous cost for new therapeutic ultrasound probes. Thus, we hypothesized that ultrasound with an acoustic lens might produce an efficient strategy for safe extensive drug delivery to the target lesion without damaging other normal tissues, additionally giving a cost-effective and straightforward solution.

**Figure 1:**
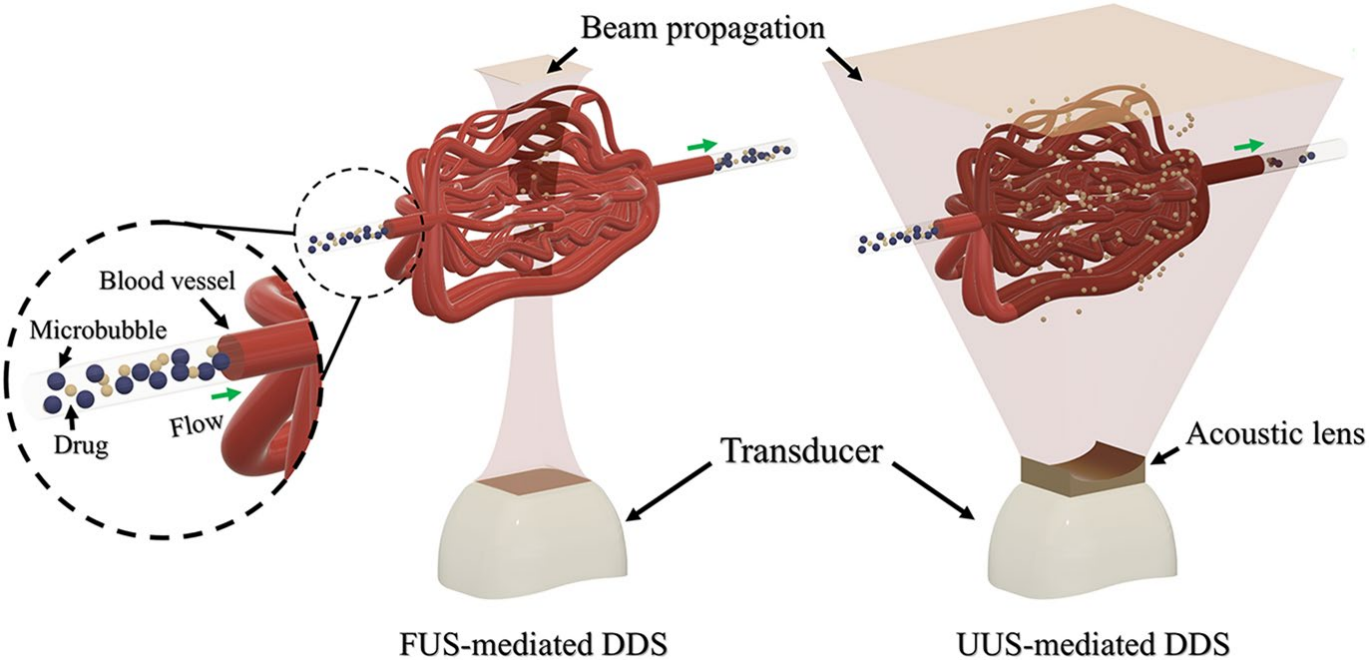
Schematic of the focused ultrasound-mediated drug delivery system (FUS-mediated DDS) and unfocused ultrasound-mediated drug delivery system (UUS-mediated DDS) with the designed acoustic lens.

This work aimed to enhance DDS effectiveness by eliminating alignment issues using broadly spreading acoustic pressure fields customized to the tumor size, as shown in Fig. 1. In addition, we tuned the acoustic pressure intensity with an acoustic lens to minimize normal tissue damage. Our approach utilizes an interchangeable and attachable compound acoustic lens in front of a conventional diagnostic ultrasonic probe and a MB injection mixed with a cytotoxic drug. The acoustic lens dimensions were designed to be suitable for ultrasound phased array probes through a commercial finite element method (FEM) simulation tool in preparation for the unfocused pressure field. To avoid unexpected damage by a widespread pressure field, we optimized the pressure field according to the tumor size and fabricated a lens to deliver the anticancer drug via uniform low-intensity ultrasound. When the pressure was sufficient to induce ultrasound-mediated MB destruction (UMMD), we studied the optimal treatment conditions for MB destruction in vitro through an optical imaging setup. Based on these results, the effects of the DDS against cancer by UUS-MBs were evaluated in a xenograft rat hepatoma model using DOX treatment.

## Results

### Acoustic characterization of the attachable divergent acoustic lens

**Figure 2:**
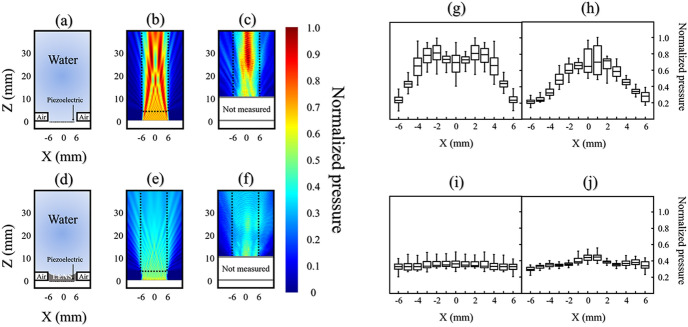
The acoustic pressure fields under either with or without the acoustic lens. Acoustic field propagations without (top) and with (bottom) the concave-shaped acoustic lens. (**a**) Schematic of the measured domain without the lens. (**b**) Simulated and (**c**) measured results of the pressure field without the lens. (**d**) Schematic of the measured domain with the lens. (**e**) Simulated and (**f**) measured results of the pressure field with the lens. The mean value distributions of (**g**) simulated and (**h**) measured acoustic pressure along one Z-axis at each X position without the lens. The mean value distributions of (**i**) simulated and (**j**) measured acoustic pressure along one Z-axis at each X position with the lens. The dotted box represents the area of interest (AOI).

Based on the results of the FEM simulation to apply the uniformly distributed pressure fields to the lesion, a concave lens with a radius of curvature (ROC) of 2.6 mm and a thickness of 4 mm was selected and fabricated. The acoustic pressure fields with and without the acoustic lens are depicted in Fig. 2a–f. When the designed concave acoustic lens was applied to the probe, the pressure field was uniformly distributed with the beam profile following the introduction of the acoustic lens compared to the pressure level without the lens. The 1 cm × 3 cm area of interest (AOI) is marked with a dashed box in Fig. 2b,c,e,f. We calculated the median values and standard deviations of each pressure distribution along the Z-axis line at each location X in the AOI. The graphs in Fig. 2g–j show each result. From the simulation results shown in Fig. 2g,i, the acoustic pressure dropped to almost half of the maximum value with the lens while uniform pressure distribution was acquired. The standard deviation of the calculated median value at each X position was reduced from 0.21 to 0.01. From the measurement data, the uniform pressure distribution showed similar results from the simulation and the standard deviation decreased from 0.18 to 0.04 (Fig. 2h,j). Thus, we confirmed that the designed concave lens could uniformly distribute the acoustic pressure in the AOI.

### In vitro microbubble destruction experiment using an unfocused uniform ultrasound beam with the lens

**Figure 3:**
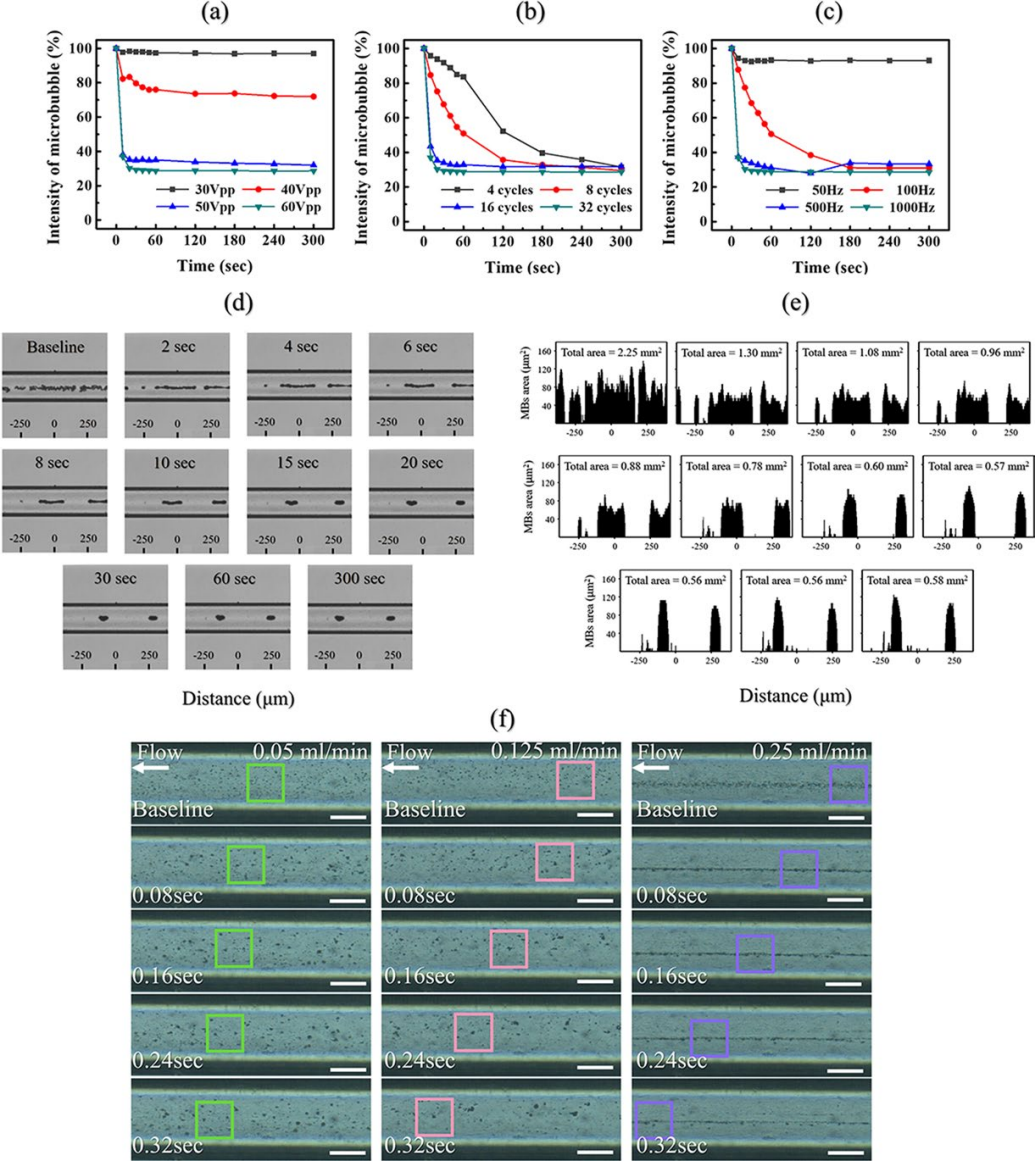
Experimental results using different variables to observe the changes in microbubbles with optical microscopy in vitro*.* Image intensity of the microbubbles versus time under several various pulse conditions, such as (**a**) voltage input (with fixed conditions of 32 pulse cycles and PRF of 1 kHz), (**b**) the number of pulse cycles (with fixed conditions of voltage input of 60 V_pp_ and PRF of 1 kHz), and (**c**) PRF (with fixed conditions of voltage input of 60 V_pp_ and 32 pulse cycles) under static conditions (n = 2). (**d**) Optical images of the microbubbles in the straight-line microchannel sonicated with the lens-attached probe applying the input voltage conditions of 60 V_pp_, 32 cycles, and PRF of 1 kHz for 5 min. No fluidic flow was applied. (**e**) Analysis of the optical images determined by calculating the pixel area of the microbubbles based on the intensity. (**f**) Optical images of the microbubble clusters with three different flow velocity setups of 0.05 ml/min, 0.125 ml/min, and 0.25 ml/min for 0.32 s of sonication in a straight line 235 μm diameter microchannel (scale bar = 100 μm).

Since acoustic attenuation through the lens material caused the pressure to decrease to 55% that of the maximum peak pressure, MB destruction must be confirmed under reduced pressure. Figure 3 shows the in vitro test results for destroying MBs by variation in the acoustic parameters such as cycle, pulse repetition frequency (PRF), and pressure amplitude. To study the threshold of optimized acoustic parameters for UMMD in the AOI, MBs were injected into the cylindrical vessel phantom with a diameter 235 μm. Various acoustic parameters were irradiated within the AOI at a distance of 40 mm, where the pressure was relatively low. The inertial cavitation of MBs, in which MBs violently collapse in a liquid, was measured through an intensity comparison baseline from the captured images. The ratios of image intensity for comparing UMMD under various cycles, PRF, and pressure amplitude are plotted in Fig. 3a–c. First, the MBs in the mimic vessel phantom were irradiated in the absence of flow conditions and measured with one variable, keeping the other conditions were fixed. Figure 3a shows that a minimum acoustic pressure of 50–60 V_pp_ is required to burst MBs. The threshold voltage (40 V_pp_) applied to the conventional ultrasonic probe with the lens to start MB collapse corresponded to 330 kPa. Figure 3b shows that the destroyed MBs depend on the number of cycles. Although all of the different numbers of cycles resulted in a 70% decrease in MB intensity within 5 min, 16–32 cycles, which burst 70% of the MBs within 20 s, were appropriate to induce a large amount of UMMD at once. Figure 3c shows that a PRF of 500–1000 Hz is needed. PRFs of 50 Hz and 100 Hz were not sufficient to induce MB rupture in bulk at one time.

Figure 3d,e present MB destruction in a straight-line microchannel with the acoustic parameters maximized (60 V_pp_ or 500 kPa, 32 pulse cycles, and a PRF of 1 kHz) for 5 min. Figure 3d shows that UMMD was saturated within 15 s, and the aggregated MBs seemed to remain stable even after 5 min. The variation in the MBs was also quantified using these captured images. We counted the number of pixels that corresponded to the contrast value of the MBs. As shown in Figure 3e, it was confirmed that 75% of the total pixel area decreased after 20 s of ultrasonic irradiation. However, after sufficient time passed after the ultrasonic irradiation, the acoustic radiation force continuously moved the aggregated MB cluster, although the total pixel area or the unaffected MBs remained unchanged. Additionally, to create a circulatory system similar to that in the human body, we observed whether the MBs responded to ultrasound in fluidic flow with the same UMMD conditions. Figure 3f presents the optical MB captured images with a time interval of 0.08 s under three different flow velocities (1.92 cm/s or 0.05 ml/min, 4.80 cm/s or 0.125 ml/min, and 9.60 cm/s or 0.25 ml/min). We applied the same acoustic conditions to the MBs in the microchannel with the flow rate setup. As a result, the MBs burst under the same conditions even at a flow rate of 0.25 ml/min (9.60 cm/s) and affected large areas, as shown in Figure 3f.

### The enhanced drug delivery system via UUS-MBs with the attachable lens

**Figure 4:**
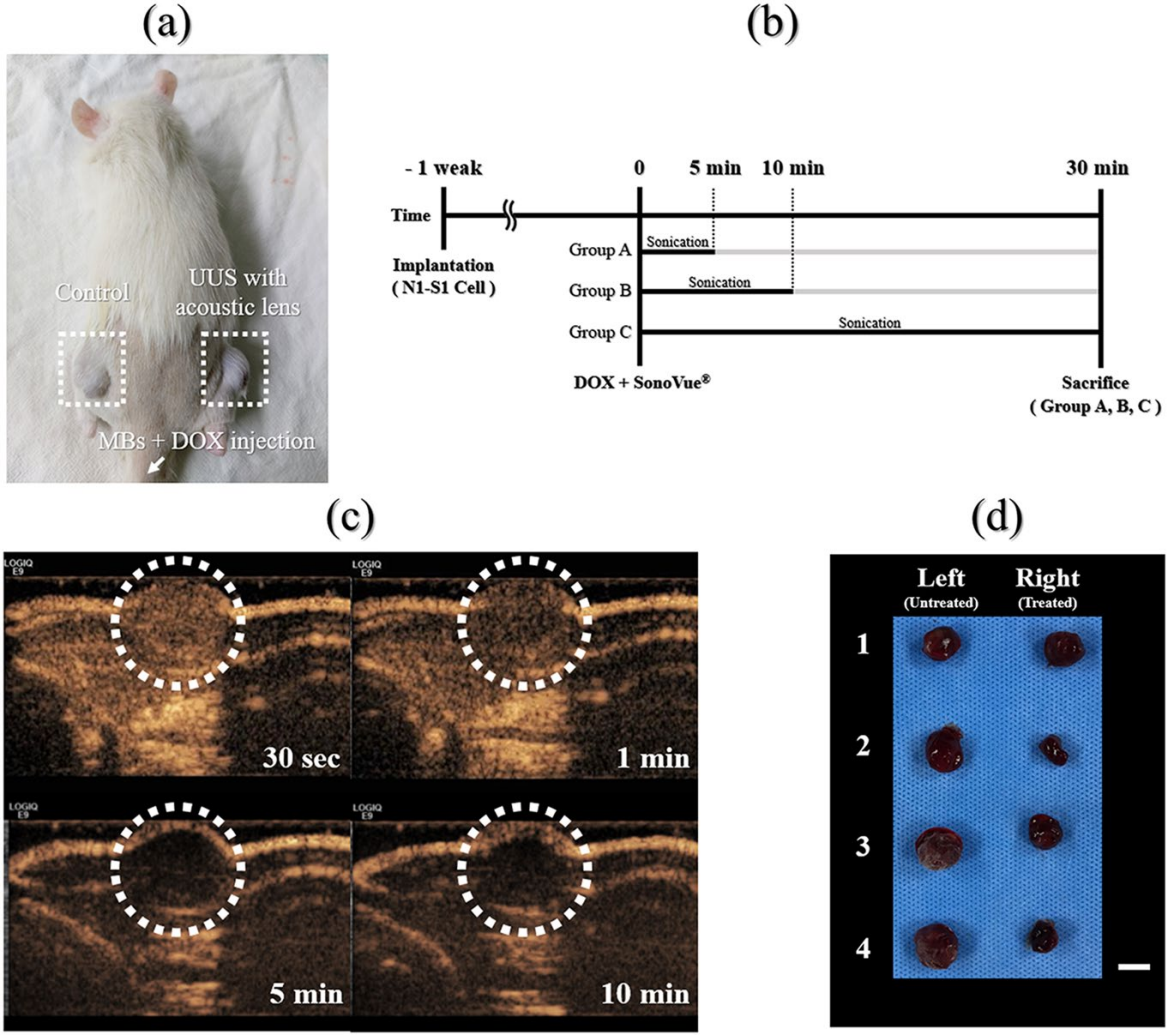
Animal experiment. (**a**) Photograph of the subcutaneous hepatoma rat model. (**b**) An illustration of the in vivo experimental setup. Doxorubicin (DOX) was firstly injected via the tail vein, and then the ultrasound treatment system was applied during each treatment time at one side. Microbubbles (MBs) were immediately injected after DOX injection. Ultrasound irradiation occurred immediately after injecting the MBs and DOX. The rats were sacrificed 30 min after the start of sonication, and the tumors were extracted to measure the DOX concentration. (**c**) Photograph of the contrast-enhanced ultrasound images after injection of the MBs. The dotted circle represents the concentration of MBs in the tumor over time. Contrast-enhanced ultrasound images were only obtained to determine the ultrasound-treated time. (**d**) Photograph of the extracted tumors from the subcutaneous hepatoma of rat models (scale bar = 1 cm).

**Figure 5:**
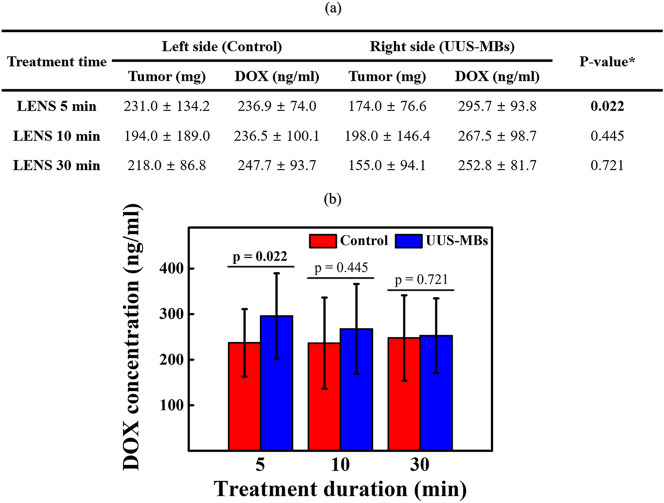
Doxorubicin concentration within the tumor in a rat xenograft hepatoma model. (**a**) Doxorubicin concentration in the subcutaneous hepatoma rat models with unfocused ultrasound-mediated microbubbles. (**b**) Graph of the change in DOX concentration according to ultrasound treatment.

To measure the enhanced chemotherapy effects using UUS-MBs with the attachable lens, we compared the DOX concentration within the tumor in a rat xenograft hepatoma model (Fig. 4). The experiment was conducted by dividing the samples into three groups according to irradiation time (5, 10, 30 min) with input voltage conditions of 60 V_pp_, 32 cycles, and a PRF of 1 kHz to find a suitable treatment time. As shown in Fig. 5, the DOX concentration increased by approximately 25% after lens treatment for 5 min (236.9 ± 74.0 ng/ml vs. 295.7 ± 93.8 ng/ml, *P* = 0.022). This DOX concentration increment drops to approximately 13% when the treatment lasts 10 min. After 30 min of UUS irradiation, the DOX concentration in the tumor was not significantly different from that in the control group. The UUS-MB group had a relatively high DOX concentration in the tumor due to the UUS-MB-induced DDS compared to the control group, but there was no significant difference in the tumor mass among any of the groups (all *P* > 0.05).

## Discussion

Many kinds of research have shown that FUS-MBs can enhance the effects of DDSs and this approach is convenient for mechanical force-triggered DDSs^23–25^. However, due to the abnormal vascular structure in the tumor and in extensive tumors, FUS-MBs have a temporal limitation in exposing all the tumor areas. This study suggested that UUS-MBs with the designed acoustic lens could be a solution for treating large tumors. We designed acoustic lens fitting to a conventional ultrasonic transducer to enhance DDS effectiveness by adjusting the pressure field for tumor size and evaluating the chemotherapeutic feasibility against cancer.

An acoustic lens, which is a mechanical aid used to adjust the acoustic propagation path, was used to control the local pressure field. Polymer-based acoustic lenses are easy to design and manufacture because acoustic propagation is controlled according to the geometric shape of the acoustic lens based on Snell's law. The lens was designed based on the geometric parameters that can uniformly irradiate the acoustic field among the results obtained by the FEM method. In this work, a polydimethylsiloxane (PDMS)-based acoustic lens was attached to a commercial diagnostic ultrasound transducer with biocompatible features. As shown in Fig. 2, ultrasound treatment with the acoustic lens, unlike the control group, could result in uniform acoustic pressure in the AOI regardless of its position. To destroy MBs in all areas of the AOI, we searched for the optimum acoustic parameters that caused the MBs to explode even in relatively low sound pressure areas.

MBs, as contrast agents for enhancing ultrasound imaging, reach the target within a few seconds, which is fast enough to be used for clinical diagnostics. Moreover, we can use MBs to induce high mechanical stress upon their collapse. This shock wave can generate locally high pressure and temperature, which is called inertial cavitation. Many kinds of research on UMMD have demonstrated that ultrasound-mediated MBs can increase the effectiveness of DDSs and induce blood–brain barrier opening via this inertial cavitation^26–28^. Thus, we similarly determined MBs as sonosensitizers to enhance chemotherapy and chose a commercial product, SonoVue (Bracco, Millan, Italy), which is widely used in the clinic. Since the effects and characteristics of MBs are not fully understood, their behavior under several ultrasound conditions must be investigated for use as the safest sonosensitizer in DDSs. Some previous studies have utilized a real-time optical microscope to monitor only a single MB or a few MBs^29–31^. However, it is essential to observe that many MBs interact with mechanical waves because a high dose of MBs is injected in the clinic. Very few experiments have been conducted to explore the interaction between a relatively large amount of MBs and ultrasound, as the limited number of MBs tested experimentally could limit their further applications. Therefore, we adopted microfluidic technology to construct an artificial blood vessel structure, and this confined channel could provide a similar in vivo environment to test the interaction between the large number of MBs and several different ultrasonic excitation conditions. This experiment was also conducted under physiologically realistic fluidic conditions compared to the in vivo circulation system.

Real-time optical imaging measurements confirmed that UUS-MBs with an acoustic lens at 60 V_pp_ for 32 cycles and a PRF of 1 kHz for 5 min could induce UMMD to promote the DDS even in fluidic conditions. It was also verified that the interaction time between the MBs and ultrasound is crucial for the DDS. First, in vitro experiments under static conditions were conducted for various pulse cycle numbers, PRFs and voltage inputs to find the optimal acoustic parameters for inertial cavitation, as shown in Fig. 3. Abrupt inertial cavitation (at a voltage of 50–60 V_pp_, 16–32 pulse cycles, or a PRF of 500–1000 Hz) exhibited promising effects, as determined by a reduction in the intensity of microbubbles compared to stable cavitation (a voltage input of 30 V_pp_ or a PRF of 50 Hz) and slow inertial cavitation (a voltage of 50–60 V_pp_, 4–8 pulse cycles or a PRF of 100 Hz). The optimal conditions placed a relatively strong acoustic radiation force on the microbubbles within 20 s, thereby disrupting the microbubble shells. Although the maximum pressure conditions were applied to the aggregated MBs to clean them thoroughly, it was hardly possible to remove the remaining MBs. We think that secondary Bjerknes force under the fixed acoustic pressure field prevented the static aggregated MBs from rupturing because the aggregated MBs generated a so-called cushioning effect so that they can mitigate each other's vigorous oscillation. On the other hand, MB aggregation and saturation phenomena decreased under fluidic conditions, but the effects of the ultrasonic waves appeared to be different from those under static conditions. This phenomenon resulted from an insufficient amount of time to see the interaction of ultrasound and flowing MBs within the observation area. This suggests that the long-term interaction between the MBs and ultrasound will be crucial in stimulating nearby tissue structures by DDS with ultrasound-mediated MBs. As one strategy to address this issue, our ultrasound system using the acoustic lens might serve as an exclusive approach that provides sufficient time to destroy moving MBs in the tumor.

Our ultrasound treatment system is capable of diverging pressure fields, and the acoustic lens can increase drug delivery effectiveness by providing an ultrasound irradiation time to MBs. The fabricated interchangeable and attachable acoustic lens demonstrated a low and uniformly acoustic pressure in AOI and optimized MB destruction in vitro experiment, which was at 60 Vpp for 32 cycles and a PRF of 1 kHz for 5 min. Based on these results, the effects of the DDS were evaluated in a xenograft rat hepatoma model using DOX treatment when the ultrasound treatment was applied for 5 min, 10 min, and 30 min. Most MBs in rats did not show avid enhancement in the tumor after 5 min but needed the further extended treatment up to 30 min to exclude little circulating MBs effect in the rat. Therefore, we divided the treatment groups into 5 min, 10 min, and 30 min (Fig. 4). UUS-MBs with the acoustic lens can promoted the DDS in vivo compared to the control group. Since sufficient irradiation time was required in the fluidic conditions, as shown in Fig. 3f, ultrasonic irradiation was conducted in consideration of the MB lifetime (< 30 min). The UUS-MBs in the acoustic lens treatment group had a higher concentration of DOX in the tumor than the control group. Although all groups showed an increase of DOX concentration in ultrasound-treated tumors, only the 5-min group showed a statistically significant increase of DOX in the tumor. In terms of extracted tumor weight, there was a slightly lower weight in the tumor on the ultrasound-treated side, but the DOX concentration (ng/ml) was analyzed using the volume of solvent adjusted by tumor extract weight (Fig. 5). Therefore, the DOX concentration might be a reliable value adjusted and normalized by tumor weight.

However, there were some limitations for the interpretation in animal experiment. First, there was no data about the initial and final tumor volume measurement by using 3D imaging modality. It might be an essential factor when dividing rats into groups by random distribution. Over 1 week after tumor implantation, the tumors were only measured using calipers to check the tumor formation. Among the bilaterally implanted rats, rats who reached above 10 mm of tumor length at both sides after 7 days were only included and then randomly distributed three groups. When the size of both tumors was measured, if either side was less than 10 mm, it was not included in the study. Therefore, the experiment was started under the assumption that there was no difference in the size of bilateral tumors. In fact, the length of the protruding subcutaneous tumor did not mean the accurate tumor volume. If the tumor characteristics such as tumor necrosis or viable tumor portion as well as tumor volume be evaluated through the contrast-enhanced imaging study such as MB-induced ultrasound imaging modality, the evidence of DDS effectiveness would be more strengthened in vivo study, because it might be a critical factor for the DOX distribution in the tumor. Second, it is difficult to mention that DOX concentrations increase as the ultrasound-treated time increases. To minimize the possible differences between subjects with tumors that had undergone the ultrasound treatment and subjects with tumors not treated with the ultrasound treatment, the tumors were implanted at one side and the other side thighs of the same subject. Therefore, the intrinsic factors such as blood circulation were minimized to compare only the ultrasound effect with DOX injection in the same rats. There was a similar DOX concentration at the non-treated side (236.9 ± 74.0 and 236.5 ± 100.1 ng/ml) and higher DOX concentration at the ultrasound-treated side (295.7 ± 93.8 and 267.5 ± 98.7 ng/ml) between 5- and 10-min groups. However, there was a higher DOX concentration at the non-treated side (247.7 ± 93.7 ng/ml) and lower DOX concentration (252.8 ± 81.7 ng/ml) at the ultrasound-treated side in the 30-min group. The accurate and logical basis for explaining this phenomenon has not been found in this study. Third, the ultrasound treatment device with the attachable lens could not show tumor imaging simultaneously because of the customized ultrasound system, which did not capture real-time ultrasound images. However, adequate generation of sonication could be monitored using the uniform wave generating by the customized ultrasound device. Further development of the ultrasound treatment device should be added to the real-time imaging module for the assessment of tumor volume and physiologic characteristics. This is easily achievable because we used the conventional diagnostic ultrasonic probe with the simple detachment of the acoustic lens. Finally, the focus of the in vivo study was the difference in final DOX concentration in tumors treated with and without the ultrasound treatment system when DOX was injected through the tail vein to treat tumors on both sides (assumed to be approximately the same size) in the same rat. However, it might also be necessary to measure the DOX concentration in normal tissues to find evidence of improving drug delivery to certain cancer cells without increasing the dose to normal cells to obtain the clinical usefulness of this DDS system.

## Conclusion

In summary, our ultrasound system is capable of diverging pressure fields and is suitable for this tumor size, and using this acoustic lens can increase drug delivery effectiveness by providing sufficient ultrasound irradiation time to the MBs. Future research is required to optimize acoustic lens designs and acoustic parameters, including the duration to maximize UMMD in blood vessels. Furthermore, ongoing animal studies using our system for cancer treatment are currently being conducted to evaluate the clinical safety of our microbubble-mediated treatment approach.

## Methods

### Custom-built ultrasonic pulser system

**Figure 6:**
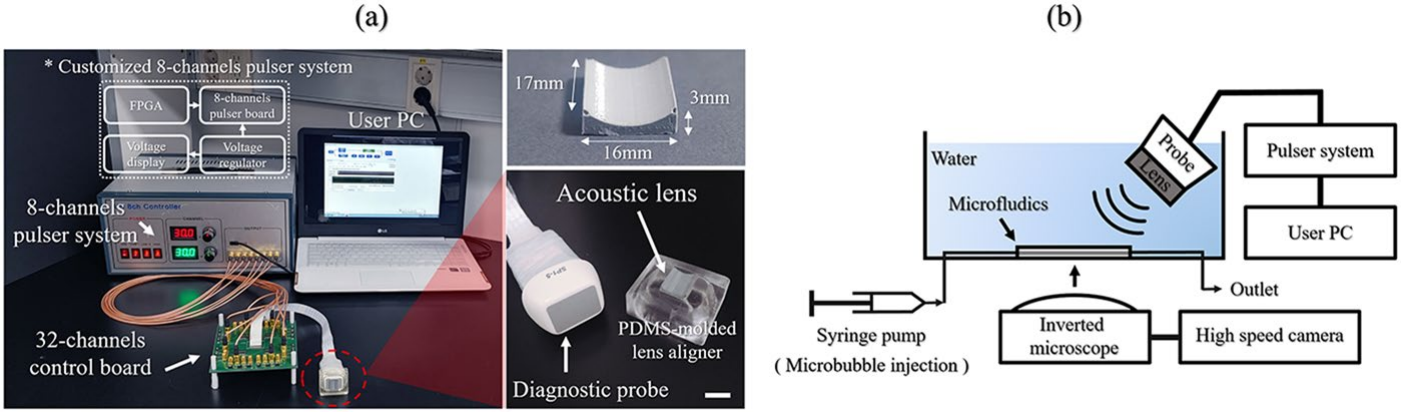
Complete ultrasonic system for the experimental study. (**a**) Photographs of the UUS-mediated DDS with the attachable acoustic lens (scalar bar = 1 cm) and the custom-built ultrasonic pulser system. (**b**) An illustration of the in vitro experimental setup. Ultrasonic waves were emitted with an incident angle of 45° to prevent a standing wave effect in the microchannel.

A photograph of the total ultrasonic system, including the attachable acoustic lens and a custom-built ultrasound pulser system, is shown in Fig. 6a. Thirty-two elements out of the 64 elements from a diagnostic phased array transducer (SP1-5; Alpinion Medical Systems, Seoul, Korea) were used to radiate ultrasound to excite the MBs. The custom-built 8-channel pulser system applied several voltage levels with a center frequency of 2 MHz to this conventional ultrasonic diagnostic probe. The pulser system was controlled by a field-programmable gate array (FPGA) on a personal computer (PC) to encode the acoustic parameters using a universal serial bus (USB) connector. We programmed the FPGA to control the ultrasonic pulse parameters, including the number of cycles, the PRF, and the probe center frequency.

### Fabrication of the concave acoustic lens

The acoustic field distribution was simulated using the finite element method (FEM) with COMSOL simulation software (COMSOL Multiphysics 5.2, COMSOL, MA, USA). A two-dimensional FEM model under continuous wave mode was used to reduce the model complexity and computational costs. The acoustic beam profiles were assessed using a hydrophone (HGL-200, Onda, CA, USA) in the presence and absence of the acoustic lens. After the hydrophone measurement, we modified the FEM model based on the experimental measurements. The polydimethylsiloxane (PDMS, Sylgard 160, Dow Corning, MI, USA) acoustic lens was replicated from a 3-D printed plastic mold designed from the final simulation.

### Optical observation of ultrasound-mediated microbubbles

The in vitro experimental setup is shown in Fig. 6b. We positioned the ultrasonic transducer with the lens slanted at 45° to the microfluidic chips' surface because we tried to eliminate the standing waves or the wave interference from the reflected surface of the water tank. Also, this helped to set the light source to directly observe the microbubble dynamics from the inverted microscope, while we kept the relative position between the channel and the incidence angle remained perpendicular. Ultrasound-mediated destruction of the MBs in the straight-line microchannel chip was monitored using an inverted microscope (IX73, Olympus, Tokyo, Japan). Optical images were captured using both an EM-CCD camera (ImagEMX2; Hamamatsu Photonics, Hamamatsu, Japan) at 70 frames per s and a high-speed camera (Phantom V2512; AMTEK VISION RESEARCH, NJ, USA) at 5000 frames per s. The PDMS microfluidic chips were fabricated using stainless steel (SUS) microneedles as a template. The microchannel (diameter = 235 μm) was connected to two tube adapters (diameter = 800 μm). Under static experimental conditions, 5.04 mg/ml ultrasound contrast agent (SonoVue, Bracco, Millan, Italy) was injected into the microchannel. Both the outlet and inlet of the microchannel were sealed with epoxy to halt the flow. Under the fluidic experimental conditions, fluid flow and MB injection were conducted using a syringe pump (Ultra 4400, Harvard Apparatus, MA, USA) and a 1-ml syringe. The dynamics of the MB destruction within the microchannel were evaluated with four different flow velocity setups. Before investigating the MB with fluid flow, the time of observation was set to 0.32 s due to the limitations of the accessible time for tracking the MBs with the fastest flow velocity setup.

The MB variation within the microchannel was quantified using custom-written image analysis code in MATLAB software (Natick, MA, USA). The captured images were loaded into MATLAB, and the RGB images were converted to grayscale intensity images. In the 235 μm-diameter microchannel (900 × 235 μm^2^), we counted the number of pixels with a cutoff value less than that of the pixel contrast value of the outer wall of the MBs. Based on this image processing setup, the relative intensity of the MBs was extracted from the captured images every 10 s or 1 min.

### Sonication by the diagnostic probe with the attachable acoustic lens with the maximal acoustic parameters

Maximal unfocused ultrasound sonication was delivered at 2 MHz with 60 V_pp_ in bursts of 32 cycles at a 1 kHz repetition time. From the AOI, the results through the hydrophone indicated that the mechanical index (MI) was 0.17 that of the average with a standard deviation of 0.03, and the I_spta_ (spatial-peak-temporal-average intensity) was 0.412 W/cm^2^ that of the average with a standard deviation of 0.124.

### Rat hepatoma model preparation and treatment

This study was approved by the Institutional Animal Care and Use Committee of the Seoul National University Hospital (IACUC; No. 18-0124-S1A1(3)) and was performed in accordance with the ARRIVE guideline. All methods were carried out in accordance with relevant guidelines and regulations. The N1-S1 (CRL-1604; ATCC, Manassas, VA) hepatoma tumor cell line was cultured in RPMI-1640 (Welsen, Daegu, Korea). The medium was supplemented with 10% fetal bovine serum and a 1% penicillin/streptomycin mixture (Gibco, Grand Island, NY, USA). Cell viability was tested with Trypan blue staining to confirm cell viability of > 90% before tumor implantation. To establish a xenografted rat tumor model, 1 × 10^7^ N1-S1 cells were collected and subcutaneously injected into the bilateral backs of Sprague-Dawley rats weighing approximately 300 g. Cyclosporine A (20 mg/kg/day; Chong Kun Dang Pharmaceutical Corp., Seoul, Korea) was subcutaneously injected 1 day before tumor implantation and for 4 days postoperatively in order to prevent spontaneous regression of the N1-S1 cells.

Over 1 week, the animals were weighed, and the tumors were measured using calipers. When the tumor length reached 10 mm after 7 days, the rats were divided into three groups: Group A (DOX + LENS 5 min, n = 10), Group B (DOX + LENS 10 min, n = 10), and Group C (DOX + LENS 30 min, n = 10) (Fig. 4a,b). Contrast-enhanced ultrasound was performed by a radiologist (J.H.K.) with 18 years of clinical experience in order to determine the treatment time for DOX delivery using a GE LOGIQ E9 Ultrasound System (GE Healthcare, Wauwatosa, WI, USA) with the following parameters: a transducer frequency of 9 MHz; a frame rate of 13 Hz; a dynamic range of 60; an MI of 0.14; a gain of 24; and a depth of 2.0 cm (Fig. 4c). DOX (3 mg/kg) was injected via the tail vein in all groups. SonoVue (0.3 mL in 0.1 ml of saline for each rat) through the tail vein and the LENS device was applied for the treatment at each treatment time. For uniform exposure of hepatoma tumor cells, the rats were positioned in the center of our customized ultrasound system. Thirty minutes after injection of the microbubbles, all rats were sacrificed, and the tumors were removed (Fig. 4d).

### Measurement of doxorubicin concentrations in the tumors

The tumors were carefully removed from the subcutaneous layer of the rat thighs. They were then used for the analysis of DOX concentration according to previously published procedures. Briefly, the tissues were homogenized in acidic alcohol (3% hydrochloride, 48.5% ethanol, 48.5% double-distilled water) with a microtissue homogenizer, and DOX was extracted for 24 h in the dark at 4 °C. The homogenates were then centrifuged at 5000 rpm for 10 min at 4 °C, and the supernatants were collected. To quantify DOX, the level of fluorescence for each of the samples was measured using liquid chromatography/tandem mass spectrometry (LC–MS/MS) [an Agilent 1260 Infinity Binary LC (Agilent Technologies, Santa Clara, CA, USA) and an API 4000 QTRAP system (AB Sciex, Framingham, MA, USA)], and the DOX concentration of each sample was calculated according to the weight of the corresponding tumor. All statistical analyses were performed using SPSS version 21.0 (SPSS, Chicago, IL, USA). The DOX concentration of each group was analyzed using the Wilcoxon-signed rank test at each treatment group.

## Data Availability

The datasets generated and/or analyzed during the current study are available from the corresponding author upon reasonable request.
